# Development of machine learning models for prediction of current and future dementia

**DOI:** 10.1371/journal.pone.0330213

**Published:** 2025-12-10

**Authors:** Wonseok Jeong, Wankyo Chung

**Affiliations:** 1 Department of Public Health Sciences, Graduate School of Public Health, Seoul National University, Gwanak-gu, Seoul, Republic of Korea; 2 Artificial Intelligence Institute, Seoul National University, Seoul, Republic of Korea; PLOS: Public Library of Science, UNITED KINGDOM OF GREAT BRITAIN AND NORTHERN IRELAND

## Abstract

Dementia is among the most distressing and burdensome health challenges in aging populations. Treatment efficacy is limited; however, early diagnosis can delay or prevent disease progression. Previous machine learning-based prediction models have limitations (e.g., they are based on clinical parameters or are not generalizable). Thus, in this study, prediction models were developed for current and future dementia solely based on demographic, socioeconomic, and health-related features. Demographic, socioeconomic, and health-related variables collected from the Korean Longitudinal Study of Ageing (KLoSA) were used to develop machine learning-based prediction models for current and future dementia with various algorithms. Two sampling strategies were used for feature selection, one based on domain knowledge and the other based on statistical testing. Hyperparameter tuning was performed using grid search with cross-validation on the training set, and model evaluation was conducted on a separate test set. In the initial no-follow-up dataset, 92 of 6,898 participants exhibited dementia. Among 6,207 participants without dementia initially, 69 developed dementia within 2 years. Linear support vector machine (SVM) and radial bias function SVM exhibited the best sensitivity for current and future dementia (79.4% and 77.7%, respectively). The SHAP (SHapley Additive exPlanations) approach improved the transparency of the model by highlighting the top ten features most strongly associated with increased dementia risk. We achieved reasonably accurate prediction results for dementia using only non-clinical features.

## 1. Introduction

Dementia is one of the most distressing and burdensome health issues in the older population [1,2]. Characterized by the loss of memory and cognitive function, dementia often leads to a decline in activities of daily living, posing a significant social and economic burden [3]. Caregivers of patients with dementia spend an average of 3.6 hours per day assisting patients with daily living activities and an additional 2.6 hours per day in overall care [4]. The prevalence of dementia in South Korea is rising rapidly due to the country’s unprecedented rate of population aging. The proportion of South Koreans aged 60 and older is projected to increase from 13.7% in 2015 to 28.6% by 2050 [5]. Consistent with this demographic shift, the number of Koreans with dementia is poised to double every 17 years from 2017 and is estimated to reach nearly 3 million by 2050 [6].

Despite numerous studies, the efficacy of dementia treatment for patients in advanced stages remains limited [7]. However, early diagnosis can delay or even prevent progression. There are different types of dementia including Alzheimer’s disease (AD), cerebrovascular dementia, benign brain tumors, frontotemporal dementia, and hypothyroidism [8]. AD, the most common form of dementia, accounts for approximately two-thirds of all dementia cases and can be delayed with early diagnosis using various treatments, such as donepezil, which promotes behavioral stabilization, preserves independence, and slows cognitive decline [9,10]. Cerebrovascular dementia is closely associated with various diseases, such as hypertension, diabetes, and cerebral infarction, thus making it preventable through risk factor management and medications. Other types of dementia that involve the thyroid or tumors can be relatively easily addressed by surgery when detected in the early stages [11]. Early identification and prevention of dementia are crucial for mitigating its global burden and enhancing quality of life in an aging society.

Recently, a growing body of literature has investigated machine learning approaches for dementia prediction [12]. However, these studies have several critical limitations, including the use of data that are not representative of the population, a focus solely on AD or general cognitive dysfunction as an outcome, utilization of only clinical datasets that are not publicly available, or predictions restricted to the current status of participants [11–14]. Despite the growing public health challenges posed by dementia and its irreversible nature, the development of accurate prediction models is an important aim.

In this study, we developed machine learning-based prediction models for both current and future dementia using demographic, socioeconomic, and health-related variables. In particular, the objectives of this study were to establish machine learning prediction models for dementia and to compare the performance of the models trained using various machine learning techniques, including lasso logistic regression (Lasso LR), ridge logistic regression (Ridge LR), linear support vector machine (Linear SVM), radial bias function support vector machine (RBF SVM), random forest (RF), and gradient boosting machine (GBM).

## 2. Materials and methods

### 2.1. Study design and data sources

Data were obtained from the seventh- and eighth-wave data (2018 and 2020) for the Korean Longitudinal Study of Ageing (KLoSA), collected every even-numbered year. The KLoSA is an ongoing longitudinal panel survey conducted by the Korea Labor Institute to collect basic data needed to devise and implement effective social and economic policies that address emerging trends related to population aging [15]. Koreans aged 45 years and older living in households were randomly selected using multistage stratified probability sampling based on province and housing type [16]. The data obtained for this study were officially approved by Statistics Korea (Approval No. 33602) and were obtained following the receipt of written informed consent from the participants in accordance with the principles of the Declaration of Helsinki. Ethical approval for the study was not required because the data were accessible to the public for scientific purposes with de-identified information [16].

### 2.2. Initial study sample

Data from the 2020 KLoSA were set as the endpoint and data from the 2018 KLoSA was set as the baseline to develop prediction models for future dementia (i.e., dementia after 2 years) among South Korean middle-aged and older adults. The initial 2018 dataset included 6,940 individuals. Participants diagnosed with dementia or mild cognitive impairment at baseline (n = 132) were excluded. Of the remaining 6,808 individuals, those who were lost to follow-up and did not participate in the 2020 survey (n = 568) were also excluded. From the resulting 6,240 individuals, we further excluded those with missing data on key covariates required for model training, including alcohol consumption, social activity, regular exercise, and depressive symptoms (n = 33). The final analytic sample consisted of 6,207 individuals who met all inclusion criteria: no baseline cognitive impairment, completion of follow-up, and availability of complete covariate data.

For the no-follow-up dataset, the 2018 KLoSA was used to develop diagnostic models for current dementia among South Korean middle-aged and older adults. Of the 6,940 individuals included in the 2018 KLoSA dataset, those with dementia, mild cognitive impairment or missing covariates were excluded. In total, 6,898 participants were included in the final dataset.

### 2.3. Feature selection

Among variables that may potentially affect dementia risk based on previous studies [17–21], chi-squared tests were used to select relevant variables for our dementia prediction models. Only variables that were statistically significant (p < 0.05) based on chi-squared tests were included in the final machine learning models after being transformed into one-hot encoded features.

Demographic variables identified in previous studies were participants’ age (55 − 65, 65 − 75, or ≥75 years), sex, and marital status (married, unmarried, and separated). Socioeconomic factors included participants’ educational level (elementary school or lower, middle school, high school, or college or higher), region (urban or rural), household income (low, lower middle, upper middle, and high), and social activity (yes or no). Social activity was defined as engaging in at least one social activity, including religious gatherings, social gatherings, leisure gatherings, non-governmental organizations, and participation in volunteer groups.

Health-related variables included the current drinking status (yes or no), regular exercise at least once a week (yes or no), current smoking status (yes or no), depression (yes or no), doctor’s diagnosis of diabetes (yes or no), doctor’s diagnosis of hypertension (yes or no), vision impairment (yes or no), hearing loss (yes or no), and eating difficulties (yes or no). Information on drinking, smoking, and exercise was self-reported in response to the question: “Are you currently engaged in these health-related behaviors on a regular basis?” Those who were either currently taking antidepressants or had self-reported experiencing depressive symptoms for at least two consecutive weeks were categorized as ‘yes’ for depression. Eating difficulties were defined as challenges caused by oral health issues. Those who frequently experienced such problems were categorized as “yes,” whereas those who did not were categorized as “no.” Finally, participants experiencing problems in their everyday lives due to vision or hearing issues were categorized as “yes” to vision impairment and hearing loss.

Dementia was an outcome of this study. Dementia was defined as any type of dementia diagnosed by a medical professional, including AD, vascular dementia, dementia with brain tumors, or hypothyroidism.

### 2.4. Model Development

**Fig 1:**
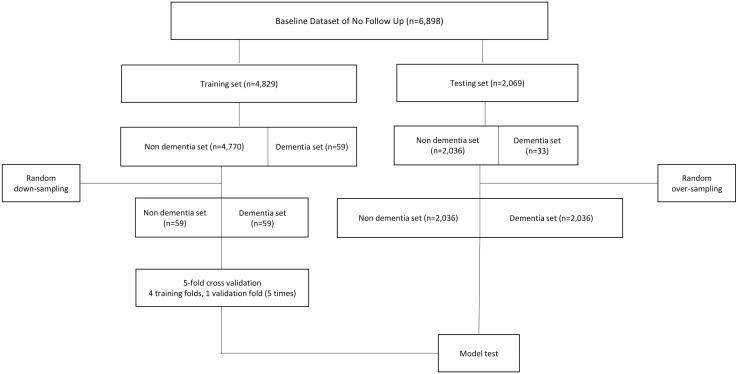
Flowchart of the prediction model development of no follow up dataset.

**Fig 2:**
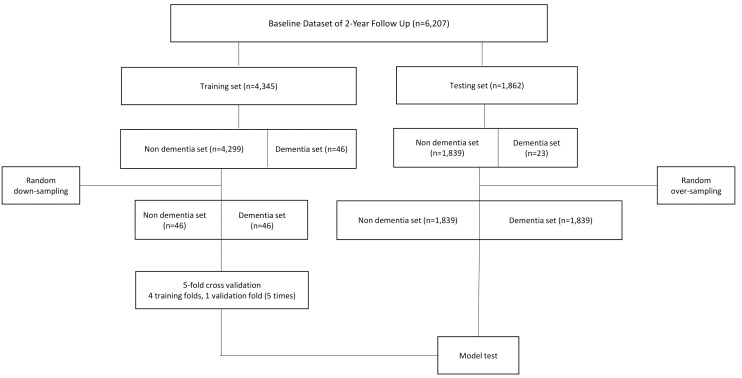
Flowchart of the prediction model development of 2-year follow up dataset.

The dataset without follow-up consisted of 6,898 participants, including 92 dementia cases (1.3%) and 6,806 non-dementia cases (98.7%). It was randomly split into training and test sets at a 7:3 ratio, using stratified sampling to preserve the overall proportion of dementia cases in both sets. Only the total number of dementia cases was considered during stratification, without preserving the detailed proportions of dementia subtypes. The resulting training set contained 4,829 participants, of which 59 were dementia cases and 4,770 were non-dementia cases. Because the non-dementia group was much larger than the dementia group, the training dataset was downsampled to address class imbalance and prevent learning bias. Specifically, 59 non-dementia cases were randomly selected from the 4,770 available, resulting in a balanced training set of 118 participants with a 1:1 ratio of dementia to non-dementia. Downsampling was chosen over oversampling to reduce the risk of overfitting caused by repeated duplication of minority class samples. The dementia group in the test dataset was randomly oversampled due to the limited number of participants with dementia, which was insufficient for a reliable evaluation of prediction performance. The 2-year follow-up dataset was subjected to the same procedure. For a detailed overview of the data processing steps and sampling strategy, refer to the flowcharts illustrating the construction of the current and 2-year dementia prediction models in Fig 1 and Fig 2, respectively.

As previously described, we first divided the dataset into separate training and test sets to ensure an unbiased assessment of the model’s performance. Then we applied 5-fold cross-validation within the training set; the training set was split into five equally sized random subsamples: one was used for validation, while the remaining four were used for training. The process was repeated five times, and the results were averaged to produce a single training performance estimate for each model. Six machine learning methods were trained: LR, Ridge LR, Linear SVM, RBF SVM, RF, GBM. The optimal hyperparameters for each machine learning method were determined through cross-validation using a grid search, with accuracy as the selection metric (Table 1). Hyperparameter tuning was performed separately for each algorithm. For ridge logistic regression, 1,000 values of L2 regularization (λ) from 0.01 to 10 were tested. The linear SVM used 1,000 logarithmically spaced values of C from about 0.03 to 750. The RBF SVM was tuned over 1,000 pairs of C (≈0.05–1000) and gamma (≈0.0087–0.0180). For random forests, we varied the number of features per split (2–12), minimum samples to split (1, 5, 10, 15), and used 1,000 to 3,000 trees with Gini impurity. The GBM model was tuned over tree depth (1–9), learning rate (0.001–0.3), leaf size (5–30), and boosting iterations (1,000–3,000). The final machine-learning models with the best hyperparameters were then used to predict dementia in the testing dataset.

**Table 1:** Optimal parameters for each machine learning model are selected through the grid search.

Dataset	Model	Optimal parameters
No Follow Up	Lasso LR	Penalty: ‘l1,’ Lamda: 0.07
	Ridge LR	Penalty: ‘l2,’ Lamda: 2.73
	Linear SVM	Cost: 0.05537025
	RBF SVM	Cost: 0.2836569, gamma: 0.01490787
	RF	n_estimators: 2000, n_features:2, min sample splits:15
	GBM	n_estimators: 1000, learning rate: 0.3, max_depth:7, min sample splits:20
2-Year Follow Up	Lasso LR	Penalty: ‘l1,’ Lamda: 0.03
	Ridge LR	Penalty: ‘l2,’ Lamda: 8.9
	Linear SVM	Cost: 0.1666947
	RBF SVM	Cost: 4.935686, gamma: 0.01214017
	RF	n_estimators: 2000, n_features:2, min sample splits:15
	GBM	n_estimators: 1000, learning rate: 0.05, max_depth:1, min sample splits:10

Note. LR: logistic regression; SVM: support vector machine; RF: random forest; GBM: gradient boosting machine.

### 2.5. Model evaluation and interpretability

Descriptive statistics were used to summarize the general characteristics of the study population, which were presented as frequencies and percentages. Chi-square tests were used to assess differences between group to select relevant variables for our dementia prediction models. Sensitivity, specificity, positive predictive value (PPV), negative predictive value (NPV), and accuracy were used as metrics to assess the performance of each machine learning model in predicting dementia. The area under the receiver operating characteristic curve (AUC) was calculated for each model to assess the overall prediction performance. To enhance model interpretability and uncover key risk factors, we applied the SHapley Additive exPlanation (SHAP) method to the best-performing model [22]. SHAP uses Shapley values to quantify the contribution of each feature, offering both global insights and case-specific explanations. This approach enables a clearer understanding of complex feature interactions, supporting model transparency and reducing the risk of overfitting. All statistical analyses were conducted using SAS 9.4 (SAS Institute Inc., Cary, NC, USA), while all machine learning approaches were performed using R (R version 4.3.2, http://www.r-project.org) and its supported packages, including caret, ROSE, glmnet, gbm, kernlab, randomForest, ggbeeswarm, and fastshap. A seed number of 124 was used for reproducibility.

## 3. Results

Table 2 presents the baseline characteristics of participants in the dataset without follow-up and comparisons of parameters between groups. Overall, 92 of the 6,898 participants (1.3%) presented with dementia and 6,806 (98.7%) did not. Only features that differed significantly between groups, determined using the chi-square test (*p* < 0.05), were included in the machine learning models. Statistically significant features included age, marital status, household income level, educational level, drinking status, regular exercise, social activity, depression, vision impairment, hearing loss, and eating difficulties. Sex is considered an important factor in social science studies; therefore, it was included in the machine learning models, regardless of the chi-squared test results. Contrary to previous results, hypertension and diabetes were not identified as significant factors in our analysis and were excluded from our models.

**Table 2:** Baseline Characteristics of the Dataset (No Follow Up).

Variables	Total		Dementia				
			Yes		No		*P*-value
Total	6,898	(100.0)	92	(1.3)	6,806	(98.7)	
**Sex**							0.1365
Male	2,925	(42.4)	32	(34.8)	2,893	(42.5)	
Female	3,973	(57.6)	60	(65.2)	3,913	(57.5)	
**Age (years)**							<0.0001
55-65	2,574	(37.3)	1	(1.1)	2,573	(37.8)	
65-75	2,029	(29.4)	14	(15.2)	2,015	(29.6)	
≥75	2,295	(33.3)	77	(83.7)	2,218	(32.6)	
**Marital status**							0.0004
Married	5,204	(75.4)	55	(59.8)	5,149	(75.7)	
Unmarried or separated	1,694	(24.6)	37	(40.2)	1,657	(24.3)	
**Household income level**							<.0001
Low	1,745	(25.3)	43	(46.7)	1,702	(25.0)	
Lower middle	1,822	(26.4)	26	(28.3)	1,796	(26.4)	
Upper middle	1,631	(23.6)	17	(18.5)	1,614	(23.7)	
High	1,700	(24.6)	6	(6.5)	1,694	(24.9)	
**Educational level**							<.0001
Elementary school or less	2,650	(38.4)	63	(68.5)	2,587	(38.0)	
Middle school	1,155	(16.7)	13	(14.1)	1,142	(16.8)	
High school	2,216	(32.1)	12	(13.0)	2,204	(32.4)	
College or over	877	(12.7)	4	(4.3)	873	(12.8)	
**Region**							0.7768
Rural area	1,712	(24.8)	24	(26.1)	1,688	(24.8)	
Urban area	5,186	(75.2)	68	(73.9)	5,118	(75.2)	
**Smoking Status**							0.4021
Smoker	535	(7.8)	5	(5.4)	530	(7.8)	
Non-Smoker	6,363	(92.2)	87	(94.6)	6,276	(92.2)	
**Drinking Status**							<.0001
Yes	2,255	(32.7)	6	(6.5)	2,249	(33.0)	
No	4,643	(67.3)	86	(93.5)	4,557	(67.0)	
**Regular Exercise**							<.0001
Yes	2,265	(32.8)	84	(91.3)	2,273	(33.4)	
No	4,541	(65.8)	84	(91.3)	4,625	(68.0)	
**Social Activity**							<.0001
Yes	5,310	(77.0)	72	(78.3)	5,290	(77.7)	
No	1,588	(23.0)	20	(21.7)	1,516	(22.3)	
**Depression**							0.0005
Yes	248	(3.6)	24	(26.1)	224	(3.3)	
No	6,650	(96.4)	68	(73.9)	6,582	(96.7)	
**Hypertension**							0.3597
Yes	179	(2.6)	1	(1.1)	178	(2.6)	
No	6,719	(97.4)	91	(98.9)	6,628	(97.4)	
**Diabetes**							0.693
Yes	114	(1.7)	2	(2.2)	112	(1.6)	
No	6,784	(98.3)	90	(97.8)	6,694	(98.4)	
**Vision Impairment**							<.0001
Yes	1,455	(21.1)	59	(64.1)	1,396	(20.5)	
No	5,443	(78.9)	33	(35.9)	5,410	(79.5)	
**Hearing Loss**							<.0001
Yes	330	(4.8)	26	(28.3)	304	(4.5)	
No	6,568	(95.2)	66	(71.7)	6,502	(95.5)	
**Eating Difficulties**							<.0001
Yes	1,225	(17.8)	49	(53.3)	1,176	(17.3)	
No	5673	(82.2)	43	(46.7)	5630	(82.7)	

Table 3 presents the baseline characteristics and comparison of parameters between groups for the 2-year follow-up dataset. Of 6,207 participants who initially did not have dementia, 69 (1.1%) developed dementia within 2 years, while the remaining 6,138 (98.9%) did not. Along with all features chosen from the no-follow-up dataset, region was included in the 2-year prediction models because it differed significantly between groups.

**Table 3:** Baseline Characteristics of the Dataset (2-Year Follow Up).

Variables	Total		Dementia				
			Yes		No		*P*-value
Total	6,207	(100.0)	69	(1.1)	6,138	(98.9)	
**Sex**							0.1333
Male	2,620	(42.2)	23	(33.3)	2,620	(42.7)	
Female	3,587	(57.8)	46	(66.7)	3,587	(58.4)	
**Age (years)**							<0.0001
55-65	2,424	(39.1)	1	(1.4)	2,423	(39.5)	
65-75	1,896	(30.5)	8	(11.6)	1,888	(30.8)	
≥75	1,887	(30.4)	60	(87.0)	1,827	(29.8)	
**Marital status**							<.0001
Married	4,736	(76.3)	39	(56.5)	4,775	(77.8)	
Unmarried or seperated	1,402	(22.6)	30	(43.5)	1,432	(23.3)	
**Household income level**							<.0001
Low	1,486	(23.9)	31	(44.9)	1,455	(23.7)	
Lower middle	1,663	(26.8)	20	(29.0)	1,643	(26.8)	
Upper middle	1,482	(23.9)	11	(15.9)	1,471	(24.0)	
High	1,576	(25.4)	7	(10.1)	1,569	(25.6)	
**Educational level**							<.0001
Elementary school or less	2,277	(36.7)	55	(79.7)	2,222	(36.2)	
Middle school	1,049	(16.9)	7	(10.1)	1,042	(17.0)	
High school	2,080	(33.5)	7	(10.1)	2,073	(33.8)	
College or over	801	(12.9)	0	(0.0)	801	(13.0)	
**Region**							0.0123
Rural area	1,536	(24.7)	26	(37.7)	1,510	(24.6)	
Urban area	4,671	(75.3)	43	(62.3)	4,628	(75.4)	
**Smoking Status**							0.5302
Smoker	485	(7.8)	4	(5.8)	481	(7.8)	
Non-Smoker	5,722	(92.2)	65	(94.2)	5,657	(92.2)	
**Drinking Status**							<.0001
Yes	2,102	(33.9)	7	(10.1)	2,095	(34.1)	
No	4,105	(66.1)	62	(89.9)	4,043	(65.9)	
**Regular Exercise**							0.0002
Yes	2,118	(34.1)	9	(13.0)	2,109	(34.4)	
No	4,089	(65.9)	60	(87.0)	4,029	(65.6)	
**Social Activity**							<.0001
Yes	4,927	(79.4)	26	(37.7)	4,901	(79.8)	
No	1,280	(20.6)	43	(62.3)	1,237	(20.2)	
**Depression**							0.0005
Yes	186	(3.0)	7	(10.1)	179	(2.9)	
No	6,021	(97.0)	62	(89.9)	5,959	(97.1)	
**Hypertension**							0.1661
Yes	166	(2.7)	0	(0.0)	166	(2.7)	
No	6,041	(97.3)	69	(100.0)	5,972	(97.3)	
**Diabetes**							0.2851
Yes	100	(1.6)	0	(0.0)	100	(1.6)	
No	6,038	(97.3)	69	(100.0)	6,107	(99.5)	
**Vision Impairment**							<.0001
Yes	1,209	(19.5)	31	(44.9)	1,178	(19.2)	
No	4,998	(80.5)	38	(55.1)	4,960	(80.8)	
**Hearing Loss**							<.0001
Yes	243	(3.9)	13	(18.8)	230	(3.7)	
No	5,964	(96.1)	56	(81.2)	5,908	(96.3)	
**Eating Difficulties**							<.0001
Yes	1,016	(16.4)	32	(46.4)	1,016	(16.6)	
No	5191	(83.6)	37	(53.6)	5191	(84.6)	

**Fig 3:**
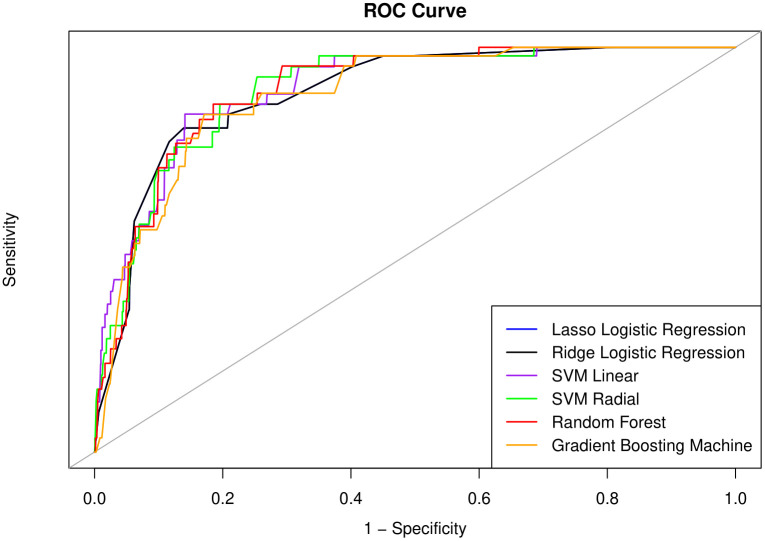
Receive operating characteristic (ROC) curve for no follow up dataset.

**Fig 4:**
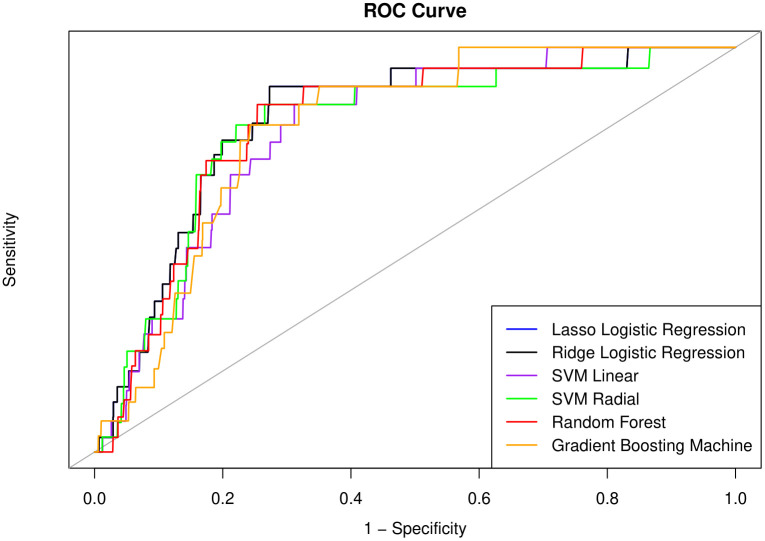
Receive operating characteristic (ROC) curve for 2-year follow up dataset.

The performances of the machine learning models on the two test datasets are summarized in Table 4. For the non-follow-up test dataset, the linear SVM presented the highest sensitivity (79.4%), whereas the RF presented the highest PPV (84.9%) and accuracy (82.4%). The RBF SVM showed the highest sensitivity (77.7%) and NPV (78.3%), whereas the RF outperformed the other models in terms of specificity (85.9%), PPV (84.1%), and accuracy (80.2%) in the 2-year follow-up dataset. The AUC values are shown in Fig 3 and 4 for the no follow-up dataset and 2-year follow-up dataset, respectively. Linear SVM presented the highest AUC (89.8) in the no-follow-up dataset, and Ridge LR showed the highest AUC (82.1) in the 2-year follow-up dataset. For every metric, the models presented superior performance for the prediction of current dementia than for the prediction of 2-year dementia. To further evaluate the robustness of the reported model performance, results on the original test set without oversampling are presented in Supplementary Table 1. Overall, all metrics except for PPV and NPV remained largely consistent when compared to those obtained using the oversampled test set. The observed pattern of extremely high NPV and low PPV in the original test set is likely due to the substantial class imbalance, which limits the reliability of some performance evaluation metrics when using the raw, highly imbalanced data.

**Table 4:** Confusion matrix for prediction models (Test set).

	Model	Sensitivity	Specificity	PPV	NPV	Accuracy	AUC
**No Follow Up**	Lasso LR	79.0%	83.5%	82.7%	79.9%	81.2%	0.887
	Ridge LR	78.8%	78.7%	78.8%	78.8%	78.8%	0.895
	Linear SVM	79.4%	83.5%	82.8%	80.2%	81.4%	0.898
	RBF SVM	78.5%	86.0%	84.8%	80.0%	82.2%	0.895
	RF	78.9%	86.0%	84.9%	80.3%	82.4%	0.895
	GBM	78.9%	83.4%	82.8%	80.6%	81.7%	0.882
**2-Year Follow Up**	Lasso LR	74.4%	80.1%	79.5%	75.9%	77.6%	0.821
	Ridge LR	76.9%	77.1%	77.0%	77.0%	77.0%	0.821
	Linear SVM	77.0%	68.5%	71.0%	74.9%	72.8%	0.795
	RBF SVM	77.7%	80.8%	80.2%	78.3%	79.2%	0.803
	RF	74.5%	85.9%	84.1%	77.0%	80.2%	0.813
	GBM	74.9%	80.8%	79.6%	76.3%	77.9%	0.802

Note. LR: logistic regression; SVM: support vector machine; RF: random forest; GBM: gradient boosting machine; PPV: positive predictive value; NPV: negative predictive value; AUC: area under ROC curve.

**Fig 5:**
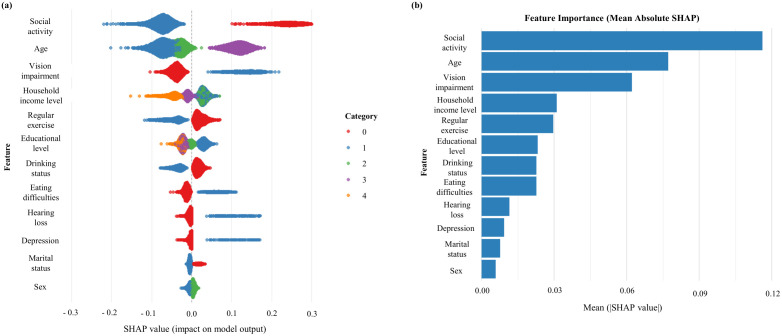
The relative importance of features for predicting current dementia.

**Fig 6:**
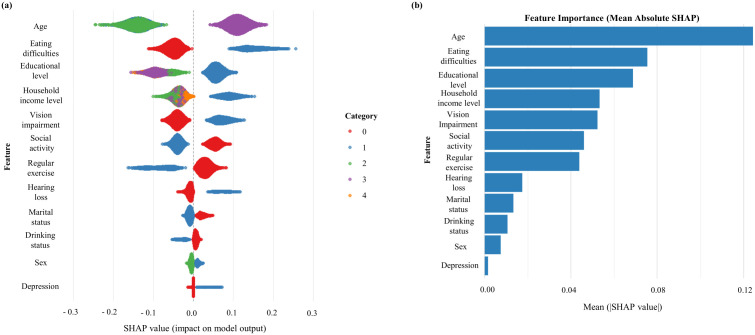
The relative importance of features for predicting future dementia.

To further interpret the results of our machine learning models, SHapley Additive exPlanations (SHAP) values were calculated to rank input variables based on their contribution to dementia prediction. Variables are displayed in descending order of importance according to their mean absolute SHAP values, which are shown on the y-axis of the summary plots. In Figs 5(a) and 6(a), each dot represents a single observation, with SHAP values plotted on the x-axis and color gradients indicating the corresponding feature values. Positive SHAP values reflect increased model output toward predicting dementia, while negative values indicate the opposite. Figs 5(b) and 6(b) display mean SHAP values as bar charts, where longer bars denote stronger average contributions to the prediction. For the current dementia model, the most influential predictors were social activity, age, vision impairment, household income level, and regular exercise. The likelihood of dementia was higher among individuals with lower levels of social and physical activity, older age, impaired vision, and lower income. In the future dementia prediction model, the top-ranked predictors included age, eating difficulties, educational level, vision impairment, and household income level. Similarly, dementia risk increased with older age, presence of eating difficulties, lower education, impaired vision, and reduced household income. For detailed variable definitions and coding schemes used in the SHAP analysis, please refer to Supplementary Table 2.

## 4. Discussion

In this study, we developed machine learning models to predict both current and future dementia using data from the 2018 and 2020 waves of the Korean Longitudinal Study of Aging (KLoSA). Dementia was defined based on physician diagnosis and included Alzheimer’s disease, vascular dementia, dementia associated with brain tumors, and hypothyroidism. The models were trained using only demographic and socioeconomic variables while still demonstrated promising performances. For the no-follow-up dataset, the linear SVM presented the highest sensitivity (79.4%) and AUC (0.898), GBM presented the highest NPV (80.6%), and RF presented the highest specificity (86.0%), PPV (84.9%), and accuracy (82.4%). For the 2-year follow-up dataset, RBF SVM presented the highest sensitivity (77.7%) and NPV (78.3%), RF presented the highest specificity (85.9%) and PPV (84.1%), and Lasso and Ridge LR presented the highest AUC (0.821).

In this study, the best model for each metric differed slightly, and there was no single prominent machine-learning technique for the diagnosis of dementia. Hence, the choice of model can vary based on different cutoffs and the perceived importance of parameters in a given context. We recommend using SVM models, since they presented the best performance in terms of sensitivity (no follow-up: linear SVM (79.4%); 2-year follow-up (RBF SVM): (77.7%)). Sensitivity, defined as the ratio of true positives to the sum of true positives and false negatives, measures the model’s ability to correctly identify patients with a disease [23]. Given the absence of a definitive treatment for late-stage dementia, the consequences of misclassifying dementia cases as non-dementia are more severe than the reverse. For the 2-year follow-up model, we strongly recommend using the RBF SVM, as it minimizes the risk of misclassification when predicting future dementia.

Previous studies have used machine learning to predict dementia by focusing on transitions from unimpaired cognition to AD and mild cognitive impairment or the conversion from mild cognitive impairment to AD [24,25,26]. In these studies, other types of dementia and individuals without initial cognitive impairment were excluded, creating significant limitations in clinical applications. Our prediction models, however, include more diverse forms of dementia, which can support effective patient stratification in clinical trials. Furthermore, unlike previously developed models, our dementia prediction models are applicable beyond clinical settings. According to a systematic review of machine learning methods for dementia prediction, most earlier models utilized neuroimaging, clinical voice recording, or clinical variable modality data [12]. These data sources are typically difficult for the general population to access and interpret, restricting the usability of such models to trained clinicians. For instance, magnetic resonance imaging (MRI), while frequently used, is not readily accessible to patients. Similarly, clinical assessments like the Mini-Mental State Examination (MMSE) and the Montreal Cognitive Assessment (MoCA) require administration by healthcare professionals. In contrast, our prediction models are based entirely on basic demographic, socioeconomic, and health-related variables. This approach ensures broader accessibility and interpretability, even in non-clinical settings. Because our models include modifiable socioeconomic factors, they can empower individuals at high risk of dementia to adopt healthier behaviors. These may include quitting smoking, reducing alcohol consumption, using assistive devices for vision and eating, and participating in regular physical or social activities. Although some predictors, such as age and sex, are immutable, and others, such as economic status and educational level, are difficult to change, individuals with these risk factors can still benefit from targeted support and tailored interventions.

In terms of performance, our models outperformed most of previously developed dementia prediction models that relied on MRI and clinical datasets. Compared with the accuracy of a previous machine learning dementia prediction model (i.e., 0.739 accuracy), our RF model exhibited a higher accuracy of 82.4%, even without official medical test scores [11]. Additionally, a systematic review reported that the average accuracy of studies using MRI as a neuroimaging technique was 74.5%, which is also significantly lower than our results [26]. Hence, even in non-clinical scenarios, individuals can easily assess their risk of dementia without visiting medical institutions, thereby substantially reducing the global economic burden and improving quality of life. Finally, given the limited treatment options available after the onset of dementia, methods for early detection and prevention are critical [7]. Yet, most previous studies have focused on diagnosing the current state of dementia using machine learning techniques [27,28]. Our models differ by aiming to predict future dementia risk, enabling proactive intervention for individuals who currently show no symptoms but are likely to develop the condition within two years. While one prior study also attempted a two-year prediction window, it achieved only 69% sensitivity using MRI alone and reached 83% sensitivity only when combining MRI with neuropsychological data [29]. Furthermore, that study included individuals with MCI at baseline and defined outcomes based on progression to progressive MCI, which differs from our broader risk-based prediction approach.

Our study had several limitations. The study population used for the training dataset was relatively small. Although the numbers of participants in the two initial datasets were 6,898 and 6,207, downsampling was performed to avoid overfitting. Limited external validation also limits the generalizability of our findings, particularly to other ethnicities. In addition, the prediction of future dementia was restricted to 2 years owing to the constraints of our dataset. Another important limitation concerns the feature selection process. We used a two-step approach involving initial manual selection based on clinical relevance, followed by statistical selection using chi-square tests. Although this approach aimed to enhance interpretability and ensure relevance, it may still introduce bias due to subjective judgment in the manual step and potential limitations of the statistical method in capturing complex relationships. Finally, our analysis did not differentiate among dementia subtypes due to limitations in the available data, which restricts the clinical specificity of our findings.

Despite these limitations, our study had several strengths. First, the KLoSA is conducted by a national institution based on random cluster sampling; therefore, the data are more reliable and representative than are data from private institutions. Second, despite the relatively small final sample sizes, cross-validation and comparisons among the five different machine learning techniques supported the reliability of our final models for predicting dementia. Third, this was one of few studies to predict both current and future dementia using machine learning techniques. Given the incurable yet preventable nature of dementia, the foresight provided by predictive models allows for necessary proactive management and other preventive measures. Finally, our models exhibit broad applicability and can be used both within and outside of clinical settings.

In conclusion, we developed machine learning-based prediction models for dementia and compared the efficiency of the algorithms using various metrics, including sensitivity, specificity, PPV, NPV, accuracy, and AUC. No single model stood out; however, SVM-based models outperformed other algorithms in terms of sensitivity and were most appropriate in our context.

Consent Statement: The data obtained for this study were officially approved by Statistics Korea (Approval No. 33602) and were obtained following the receipt of written informed consent from the participants in accordance with the principles of the Declaration of Helsinki.

## Supporting information

S1 Table Confusion matrix for prediction models (Test set without oversampling).
(DOCX)

S2 Table Features classification criteria.
(DOCX)
