# Supplementary material for: Development of machine learning models for prediction of current and future dementia
Source: PLoS One. 2025 Dec 10;20(12):e0330213. doi: 10.1371/journal.pone.0330213 (PMC12694792; doi:10.1371/journal.pone.0330213)
Supplement: S1 Table — Confusion matrix for prediction models (Test set without oversampling). (DOCX) [file pone.0330213.s001.docx]

| **Supplementary Table 1. Confusion matrix for prediction models (Test set without oversampling)** | | | | | | | |
| --- | --- | --- | --- | --- | --- | --- | --- |
|  | **Model** | **Sensitivity** | **Specificity** | **PPV** | **NPV** | **Accuracy** | **AUC** |
| **No Follow Up** | Lasso LR | 79.0% | 81.8% | 5.9% | 99.6% | 79.0% | 0.881 |
|  | Ridge LR | 78.8% | 78.7% | 5.6% | 99.5% | 78.8% | 0.897 |
|  | Linear SVM | 79.4% | 81.8% | 6.0% | 99.6% | 79.4% | 0.891 |
|  | RBF SVM | 78.5% | 84.8% | 6.0% | 99.7% | 78.6% | 0.889 |
|  | RF | 78.7% | 84.8% | 6.1% | 99.6% | 78.8% | 0.89 |
|  | GBM | 76.8% | 84.8% | 5.6% | 99.7% | 76.9% | 0.874 |
| **2-Year Follow Up** | Lasso LR | 74.4% | 82.6% | 3.9% | 99.7% | 74.5% | 0.817 |
|  | Ridge LR | 76.9% | 70.0% | 4.0% | 99.6% | 77.0% | 0.83 |
|  | Linear SVM | 77.0% | 68.5% | 3.6% | 99.5% | 72.8% | 0.804 |
|  | RBF SVM | 77.7% | 82.6% | 4.4% | 99.7% | 77.7% | 0.812 |
|  | RF | 74.8% | 87.0% | 4.1% | 99.88% | 75.0% | 0.823 |
|  | GBM | 73.7% | 82.6% | 3.8% | 99.7% | 74.0% | 0.81 |

Note. LR: logistic regression; SVM: support vector machine; RF: random forest; GBM: gradient boosting machine; PPV: positive predictive value; NPV: negative predictive value; AUC: area under ROC curve
