# Supplementary material for: Development of machine learning models for prediction of current and future dementia
Source: PLoS One. 2025 Dec 10;20(12):e0330213. doi: 10.1371/journal.pone.0330213 (PMC12694792; doi:10.1371/journal.pone.0330213)
Supplement: S2 Table — Features classification criteria. (DOCX) [file pone.0330213.s002.docx]

| **Supplementary Table 2. Features Classification Criteria** | | |
| --- | --- | --- |
| **Features** | **Features Classification Criteria** |  |
| Sex | 0: Male, 1: Female | |
| Age | 1: 55-65, 2: 65-67, 3: ≤75 | |
| Household income level | 1: Low, 2: Lower middle, 3: Upper middle, 4: High | |
| Educational level | 1: Elementary school or less, 2: Middle school, 3: High school, 4: College or over | |
| Marital status | 0: Married, 1: Unmarried or separated | |
| Social activity | 0: No, 1: Yes | |
| Drinking status | 0: No, 1: Yes | |
| Eating difficulties | 0: No, 1: Yes | |
| Hearing loss | 0: No, 1: Yes | |
| Depression | 0: No, 1: Yes | |
| Vision Impairment | 0: No, 1: Yes | |
| Regular Exercise | 0: No, 1: Yes | |
